# Quantitative Seismic Damage Assessment of Resilient Concrete Columns Using Drift Ratio-Based Fractal Dimension

**DOI:** 10.3390/ma17235850

**Published:** 2024-11-28

**Authors:** Bunka Son, Ganggang Li, Zhiwei Luo, Yuping Sun

**Affiliations:** 1 Graduate School of System Informatics, Kobe University, 1-1 Rokkodai-cho, Nada, Kobe 657-8501, Japan; wsun54725@gmail.com (B.S.); luo@gold.kobe-u.ac.jp (Z.L.); 2 Graduate School of Engineering, Kobe University, 1-1 Rokkodai-cho, Nada, Kobe 657-8501, Japan; 191t701t@stu.kobe-u.ac.jp

**Keywords:** seismic damage assessment, resilient concrete column, box-counting algorithm, fractal dimension, transient drift ratio

## Abstract

The objective of this paper is to develop assessment models to quantitatively evaluate the seismic damage caused to resilient concrete columns intended for buildings located in strong-earthquake-prone regions such as Japan and China. The proposed damage assessment models are based on the fractal analysis of crack patterns on the surface of damaged concrete columns and expressed in the form of a fractal dimension (FD) versus transient drift ratio relationship. To calibrate the proposed damage assessment models, a total of eighty images of crack patterns for eight concrete columns were utilized. All the columns were reinforced by weakly bonded ultra-high-strength (WBUHS) rebars and tested under reversed cyclic loading. The experimental variables covered the shear span ratio of the column, the concrete strength, the axial load ratio, and the amount of steel in the WBUHS rebars. A box-counting algorithm was adopted to calculate or derive the FD of the crack pattern corresponding to each transient drift ratio. The test results reveal that the FD is an efficient image-based quantitative indicator of seismic damage degree for resilient concrete columns and correlates strongly with the transient drift ratio and is subjected to the influence of the shear span ratio. The influence of the other experimental variables on the derived FDs is, if any, little. Based on the test results, a linear equation was developed to define the relationships between the FD and transient drift ratio, and a multi-linear equation was formulated to relate the transient drift ratio to the residual drift ratio, an important index adopted in current design guidelines to measure the repairability of damaged concrete structures. To further verify the efficiency of the drift ratio-based FD in seismic damage assessment, the correlation between the FD and relative stiffness loss (RSL), an indicator used to measure the overall damage degree of concrete structures, was also examined. The driven FD exhibited very strong correlation with RSL, and an empirical equation was developed to reliably assess the overall seismic damage degree of resilient concrete columns with an FD.

## 1. Introduction

Seismic damage of concrete buildings observed during recent major earthquakes [1,2,3,4,5] has made the importance of the resilience and post-earthquake restorability of concrete buildings become widely recognized in the structural engineering community from the viewpoints of the prompt re-occupancy of buildings and the quick resumption of social activities. Because the resilience and post-earthquake restorability of concrete buildings are strongly related to the repairability of damaged concrete components, the rational damage assessment of them plays an important role in the quantitative evaluation of resilience and restorability.

In the framework of a conventional strength-based design approach adopted in the modern seismic design codes [6,7,8,9], ductility factor [10] and an index proposed by Park et al. [11], which combines ductility factor and hysteretic energy dissipation capacity, have been widely used to evaluate the damage degree of damaged concrete components, and several damage assessment guidelines have been developed [12,13,14]. However, because the definition of the yield deformation is arbitrary and the yield deformation itself is also hard to be determined for damaged concrete components, these indicators are obviously not rational or suitable for post-earthquake damage assessment.

The performance-based seismic design (PBSD) approach has recently been developed and recommended in several seismic design guidelines [15,16] to overcome this deficiency inherent in the strength-based design approach. In the PBSD approach, the damage degree and restorability of damaged concrete components are assessed in terms of residual crack width and/or residual drift ratio, because these two indicators are not only directly related to the surface cracking state and the tilting degree of the damaged components, but also can be measured visually or electronically. Currently, for damaged concrete components, JBDPA [17] recommends using residual crack width to categorize the damage state, and FEMA [16] prescribes transient and residual drift ratios to classify the damage state and repairability based on the previous studies [18,19]. 

The residual crack width certainly gives a clear image of damaged concrete components, but it only represents the damage degree of the single and most significant crack. Since cracks generally occur and propagate along with deformation randomly on the surface of damaged components, a comprehensive and quantitative evaluation of restorability and repairability should take into account the crack distribution or pattern. In other words, the quantification of the surface crack pattern is indispensable.

The measurement of the residual crack width and surface crack pattern of damaged concrete components mainly relies on empirical visual inspection by structural experts. Since each component needs measurement, it usually takes a huge amount of time to obtain necessary data for defining the damage level, even for a single structure. In addition, post-earthquake inspection is accompanied with danger in most damaged structures, which tends to hinder the efficient and timely judgment of the damage state. Furthermore, because it is a visual estimation by humans, human errors are inevitable, which will affect the accuracy of the assessed damage degree.

To minimize inefficiency and inaccuracy in empirical vision-based inspections by humans, information engineering techniques have been recently adopted in damage assessment for concrete infrastructures and buildings along with recent advances in image processing and image analysis technology. Numerous studies have been conducted on the extraction of cracks and the measurement of inter-story drift ratios for damaged concrete bridges and structures [20,21,22,23,24,25,26,27,28,29,30] by using digital image correlation and the imaging processing of images taken with digital cameras. These previous studies, however, concentrated on the non-destructive detection of cracks, but did not provide any quantitative evaluation indicators for the crack patterns, much less for the propagation of cracks along with deformation. 

Fractal analysis has recently been introduced to quantitatively evaluate the crack distribution or pattern on the surface of concrete components to overcome the ambiguity inherent in the terms of minor, moderate, and major conventionally used to describe seismic damage degree. The box-counting algorithm is utilized to calculate the fractal dimension (FD) of crack patterns taken by digital cameras and then uses the driven FD to quantify the complexity and irregularity of them. Representative applications of fractal analysis for quantitatively evaluating the crack pattern of damaged concrete components inclusive of beams, columns, and walls can be found elsewhere [31,32,33,34,35]. 

Most of the previous studies on the application of fractal analysis focused on the correlation between the driven FD and the residual strength, which was defined as the ratio of the developed lateral strength under a certain level of earthquake to the maximum lateral strength of a concrete component. Since both the residual and maximum strengths of damaged concrete components are almost impossible to be measured or inferred from visual inspection and/or digital images of crack patterns, the residual strength-based FD is not a suitable candidate for post-earthquake damage assessment. Therefore, a damage assessment based on the drift ratio, which can be easily measured using digital instruments promptly and efficiently after earthquakes, needs to be developed. In addition, residual strength-based FDs were primarily calibrated against the test specimens under monotonic loading; fundamental information on the propagation of crack patterns for concrete components under reversed cyclic loading is scarce.

Objectives of this paper are (1) to provide basic data on the propagation of crack patterns, residual crack widths, and residual drift ratios of resilient concrete columns under earthquake-simulating reversed cyclic lateral loads; (2) to verify the usefulness of fractal analysis in the quantification of seismic damage for resilient concrete columns; (3) to develop drift ratio-based models for evaluating the FD from the transient drift ratio; and (4) to examine the correlation between the FD and the relative stiffness loss (RSL), an index to measure the overall damage of concrete components [10].

This paper focuses on the fractal analysis of resilient concrete columns because they are suitable for the PBSD framework. A resilient concrete column can be simply realized by utilizing weakly bonded ultra-high strength (WBUHS) rebars as longitudinal steel for concrete columns [36]. Except the six columns previously tested by the authors [37,38], two more columns were tested recently to provide additional information on the influence of the steel ratio of WBUHS rebars. These columns cover four primary structural factors with relatively wide varying ranges and can provide essential information about the influence of these factors on seismic performance and damage propagation and degree for resilient concrete columns.

## 2. Outlines of Fractal Analysis

### 2.1. Fractal Dimension

The term “fractal” or “mono-fractal” was first introduced by Mandelbrot [39] to indicate objects whose complex geometry cannot be characterized by an integer Euclidean dimension. A fractal dimension (FD) is an index for characterizing fractal patterns by quantifying their complexity as a ratio of the change in detail to the change in scale. In addition to their application in the field of structural engineering [31,32,33,34,35], fractal dimensions are also used in the medical field [40,41,42] and in geosciences [43,44,45].

### 2.2. Box-Counting Method

Several methods have been proposed for calculating FDs [39,42,46]. In this study, the box-counting method will be adopted to calculate the FDs of the crack patterns on the surface of resilient concrete columns because of its high popularity [46] and easiness of being incorporated into software dealing with image patterns extracted from digital media. 

The box-counting algorithm is based on the approach that the area of a complex object is an aggregation of smaller square boxes. In this paper, the smaller box covering the target complex object will be referred to as the element hereafter. Let the side length of the element be *r*, and the number of elements is counted to see how many of them are necessary to cover the target object. As the size or side length of the element approaches zero, the total area covered by the elements will converge to the measure of FD for the target [46]. The mathematical expression for estimating the FD can then be written as follows:

(1) $$FD=\underset{r\to 0}{\lim}\left(\frac{log(N\left(r\right))}{\log\left(1/r\right)}\right)$$

where N(r) is the total number of boxes with size *r* required to completely cover the target. In this paper, the “target” represents the crack pattern on the surface of concrete columns.

### 2.3. Procedures of the Box-Counting Algorithm

**Figure 1:**
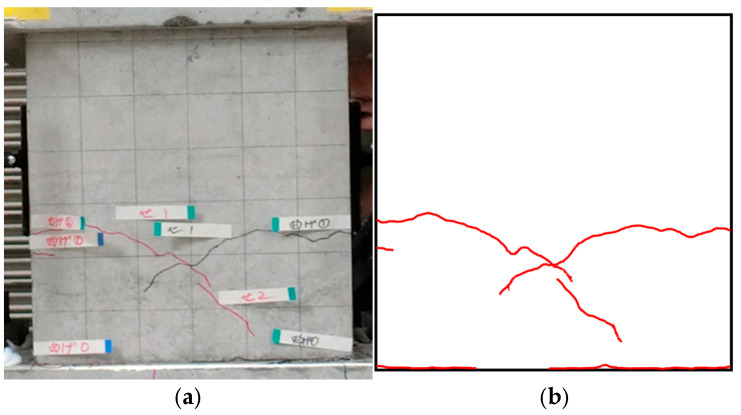
Remapping of sample image of the crack pattern taken at R = 1.0% (specimen S17N10FC50); (**a**) original; (**b**) remapped.

**Figure 2:**
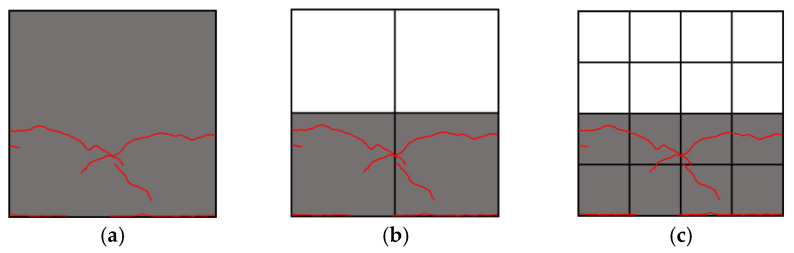

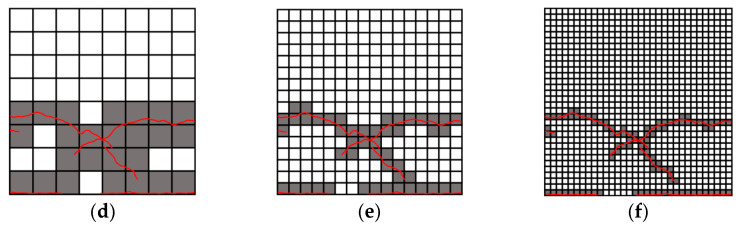
Example of dividing the target by boxes with the size r; (**a**) H; (**b**) H/2; (**c**) H/4; (**d**) H/8; (**e**) H/16; (**f**) H/32.

**Figure 3:**
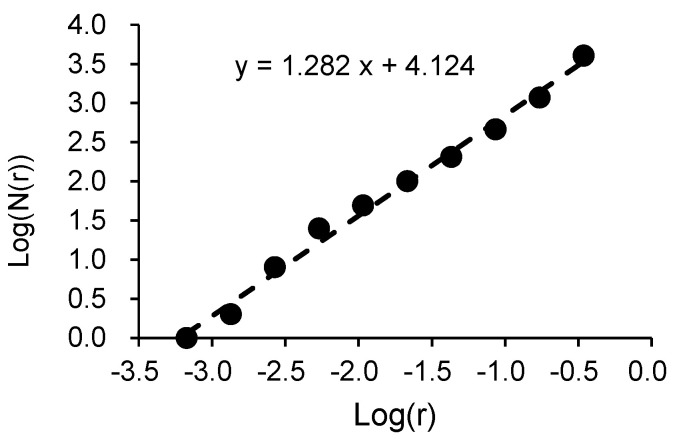
The driven FD for the exemplar image taken at R = 1.0% (specimen S17N10FC50).

As described later in the paper, cracks randomly appear on the surfaces of concrete components and propagate as the deformation in terms of drift ratio increases. The procedures for calculating the FD of the crack patterns of concrete columns corresponding to any specific drift ratio by the box-counting algorithm can be summarized as follows:

(**Step 1**) Input the original image of crack pattern taken by a high-resolution digital camera at any specific drift ratio. As an example, Figure 1a shows the original image of the crack pattern taken at the 1.0% drift ratio for specimen S17N10D06, whose details will be given later.
(**Step 2**) Track and remap the crack pattern to eliminate the influence of the initial hairline cracks caused by the dry shrinkage of concrete and the grid lines drawn on the surface, and save the remapped image (see Figure 1b) in the format of PNG. This PNG image will be referred to as the “input image” hereafter.
(**Step 3**) Give an initial value, e.g., H/2 (H is the length of the short side of the input image in pixels), to the size *r* of the square box.
(**Step 4**) Divide the input image into the boxes with the same size *r* (see Figure 2).
(**Step 5**) Count the number of boxes that cover all cracks and obtain the value of *N*(*r*) corresponding to the given *r*.
(**Step 6**) Halve the size *r* of the box as the new size *r* and repeat (**Step 4**) and (**Step 5**) to obtain the *N*(*r*) corresponding to the halved *r*.
(**Step 7**) Repeat (**Step 4**) through (**Step 6**) until the size *r* approaches approximately 1 pixel, and obtain many pairs of values for *r* and *N*(*r*) at the specific drift ratio. Examples of the driven values of *N*(*r*) corresponding to different size *r* values are shown in Table 1.
(**Step 8**) Plot *r* and *N*(*r*) in the log-log format (see Figure 3).
(**Step 9**) Following the definition by Equation (1), take the slope of the least square fit line as the FD corresponding to the specific drift. For the sample of crack pattern shown in Figure 1, its FD is then estimated as 1.282.

**Figure 4:**
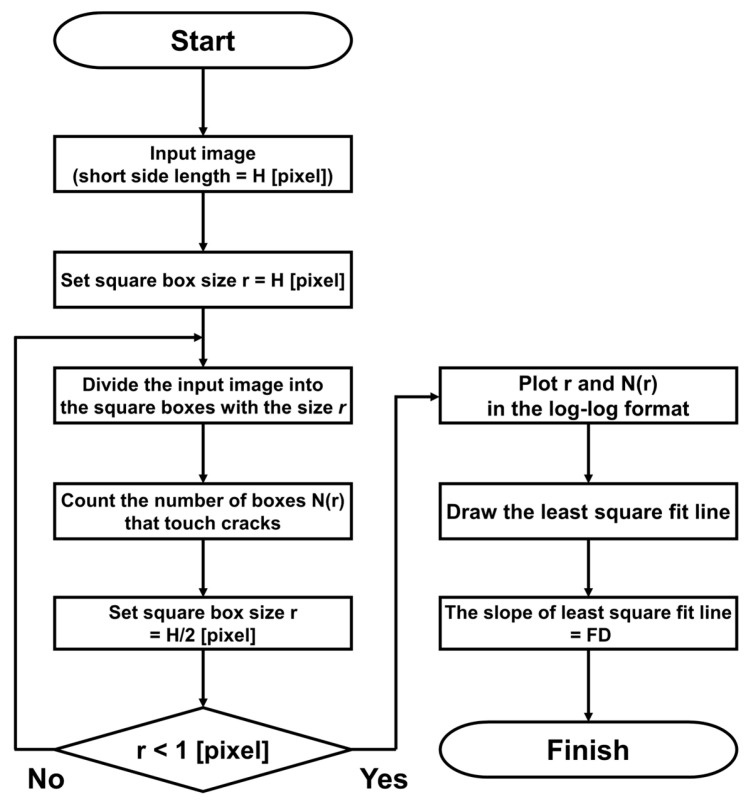
Flowchart of calculating FD using box-counting algorithm.

The procedures are also shown in the form of the flowchart in Figure 4 to help readers to utilize the box-counting algorithm. The above steps can be easily implemented in the Processing platform [47] using Java script to make the prompt and reliable estimation of the FDs for the crack pattern at any drift ratio. The remapping of the original images of crack patterns can be manually performed using Microsoft PowerPoint to minimize the influence of noise from the handwritten records and the grid lines drawn on the surfaces of the columns.

## 3. Experimental Program

### 3.1. Specimens and Material Properties

It is well known that the primary structural factors that dominate the earthquake-resisting capacity of reinforced concrete columns in accordance with modern seismic design codes include the shear span ratio of the column, the concrete strength, the axial load level, and the amount of steel in longitudinal rebars. To investigate influences of these structural factors on the seismic performance and crack propagation of resilient concrete columns, eight half-scale specimens with a 300 mm square cross-section were fabricated and tested under reversed cyclic lateral and constant axial compression. The specimens consisted of six concrete columns reinforced with eight SBPDN bars with nominal diameters of 15 mm (U15 bar) [37,38] and two concrete columns (specimens S25N10FC40 and S25N21FC40 in Table 2) reinforced with twelve U15 bars and tested recently. It is noted that the SBPDN bar is one the WBUHS bars with a specific yield strength of 1275 MPa and a bond strength of about 3.0 MPa, which is about one-fifth of a general deformed high-strength bar [48].

**Figure 5:**
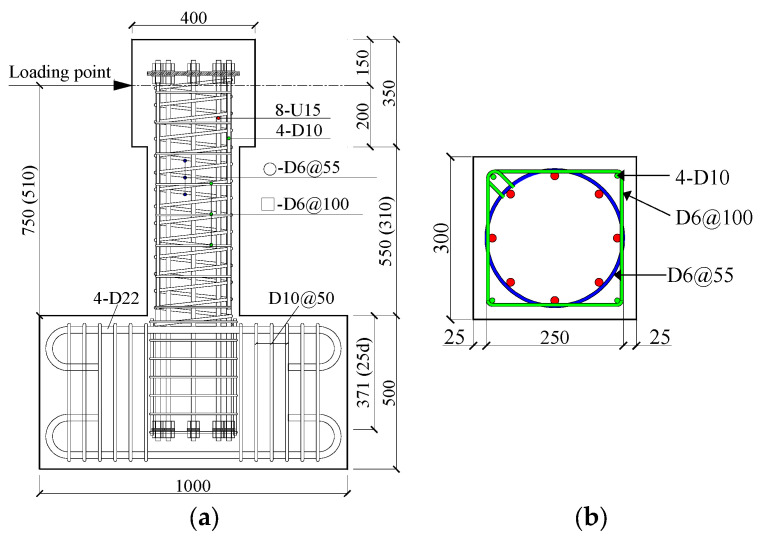
Reinforcement details and dimensions of specimens (in mm): (**a**) elevation; (**b**) section.

Experimental parameters and reinforcement details of the specimens are shown in Table 2 and Figure 5, respectively, and Table 3 summarizes the mechanical properties of the U15 bar and D6 deformed bar. As one can see from Table 2, the experimental variables were the shear span ratio (a/D = 1.7 and 2.5), the axial load ratio (*n* = 0.10 and 0.21), the concrete strength (*f_c_′* = 40 MPa and 50 MPa), and the steel ratio of SBPDN rebars (*p_g_* = 1.51% and 2.30%). The designed values of the experimental variables are well-known dominant parameters influencing seismic behavior and can cover most of the concrete columns used in middle- and high-rise buildings.

As illustrated in Figure 5, the longitudinal rebars consisted of eight or twelve SBPDN bars (U15) circularly arranged along the perimeter of the core with a diameter of 250 mm. To prevent the premature slippage of SBPDN rebars, both ends of each SBPDN rebar were anchored to a circular steel ring with thickness of 9 mm by two nuts. The transverse steels were composed of a spiral with spacing of 55 mm and square hoops with spacing of 100 mm. The D6 deformed spiral and square hoops were placed to confine the core concrete and to prevent premature spalling of the concrete shell, respectively. The square hoops were supported by four corner-placed D10 deformed bars, which were cut off at the section 10 mm away from the top surface of the foundation beam so that the D10 rebars did not directly resist axial stress induced by axial compression and moment.

Ready-mixed concrete with targeted strengths of 40 MPa and 50 MPa was used to make the specimens. Portland cement and coarse aggregates with a maximum size of 20 mm were used to make the concrete. Three standard concrete cylinders (100 mm in diameter and 200 mm in height) were prepared for each specimen, and all cylinders were cured under the same conditions as the specimens. The actual compressive cylinder strengths at the testing stages are shown in Table 2. 

### 3.2. Loading Apparatus and Instrumentations 

**Figure 6:**
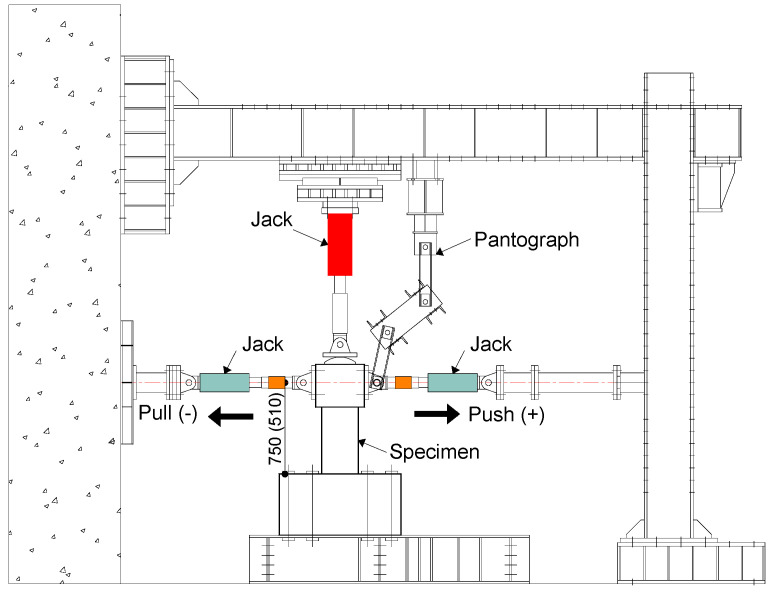
Loading apparatus.

**Figure 7:**
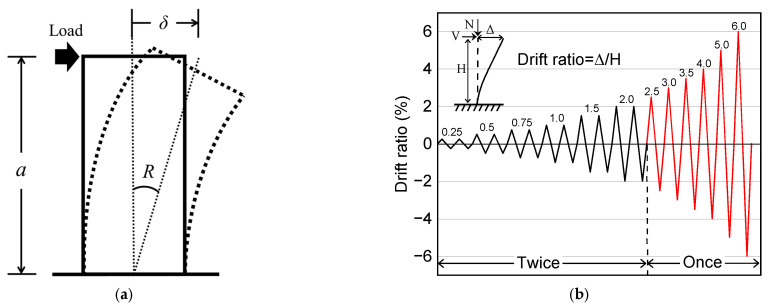
Definition of drift ratio R and loading protocol: (**a**) definition of R; (**b**) loading program.

Figure 6 illustrates the loading apparatus. As shown in Figure 6, the axial compression was applied using a 1000 kN capacity hydraulic jack before applying the cyclic lateral loading via two 300 kN capacity jacks. The reversed cyclic lateral loading was controlled by the drift ratio (R), which is the ratio of the tip lateral displacement (δ) to the shear span (*a*) of the column and is defined in Figure 7a. The loading program, which has been generally adopted in Japan, is displayed in Figure 7b. As is obvious from Figure 7b, the lateral load was applied for two complete cycles at the transient drift ratios of 0.25%, 0.5%, 0.75%, 1.0%, 1.5%, and 2.0%. Only one complete cycle was performed at the drift ratio larger than 2.5% up to 6.0%. The testing was terminated either when the column could no longer sustain axial gravity or when the drift ratio reached 6.0%, which corresponded to the stroke limitation of the jacks applying the lateral load.

The displacement transducers were installed to measure the tip lateral displacement and vertical deformation of the specimens, while a digital camera with resolution of 2160 × 3840 was used to take the original images of the cracks appearing on the surface of the columns at each transient drift ratio specified in Figure 7b. This digital camera could identify cracks with widths as small as 0.08 mm when the image was taken about 2000 mm away from the column.

## 4. Experimental Results and Discussion

### 4.1. Observed Damage and Crack Propagation

**Figure 8:**
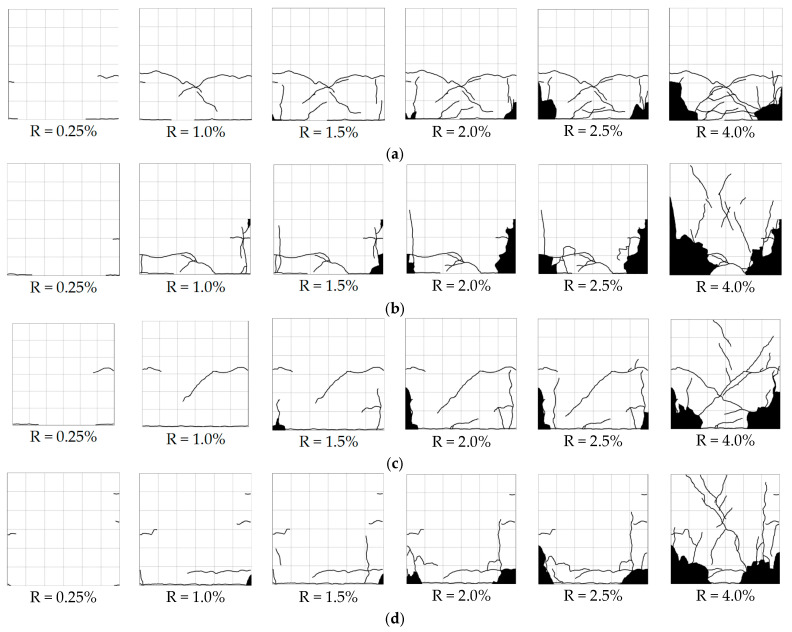
Observed propagation of cracks for specimens with a/D ratio of 1.7: (**a**) S17N10FC50; (**b**) S17N21FC50; (**c**) S17N10FC40; (**d**) S17N21FC40.

**Figure 9:**
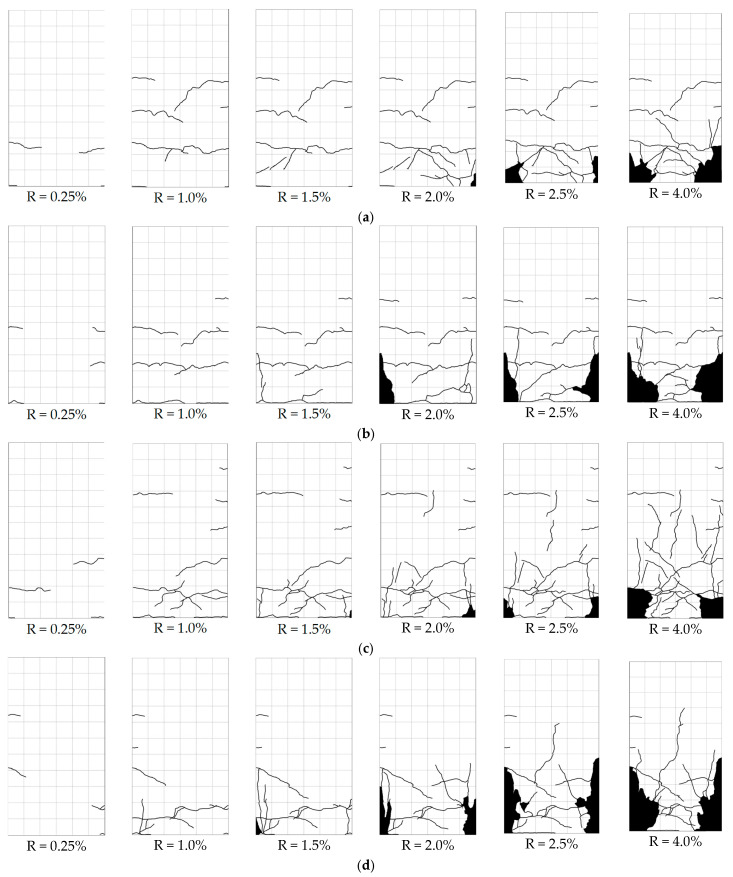
Observed propagation of cracks for specimens with a/D ratio of 2.5: (**a**) S25N10FC50; (**b**) S25N21FC50; (**c**) S25N10FC40; (**d**) S25N21FC40.

Figure 8 and Figure 9 show the propagation of the remapped crack patterns on the surface along with the drift ratio for the specimens with a/D ratios of 1.7 and of 2.5, respectively. While crack propagation was photographed and remapped at each transient drift ratio shown in Figure 6, only the crack patterns observed at transient drift ratios of 0.25%, 1.0%, 1.5%, 2.0%, 2.5%, and 4.0% are displayed because they provide sufficient information about the propagation of crack patterns for the tested columns. The black areas near the column feet shown in Figure 8 and Figure 9 represent the area of spalled-off or crushed concrete. 

Flexural cracks occurred in all specimens near the column bottom as the drift ratio reached 0.20% and became apparent at a 0.25% drift ratio regardless of the differences in the four experimental variables. The flexural cracks gradually and randomly propagated as the drift ratio increased. The longer the shear span, the wider the flexural cracks that propagated, while the higher axial load tended to limit the propagation of flexural cracks. Slight flaking of the cover concrete was observed at R = 1.0%, but obvious crushing of the cover concrete did not occur until the drift ratio reached 2.0%. The area of crushed concrete concentrated near the bottom and expanded into the column core as the drift ratio increased. No significant shear cracks were observed even at the drift ratio of 4.0%.

### 4.2. Lateral Load Versus Drift Ratio Relationships

**Figure 10:**
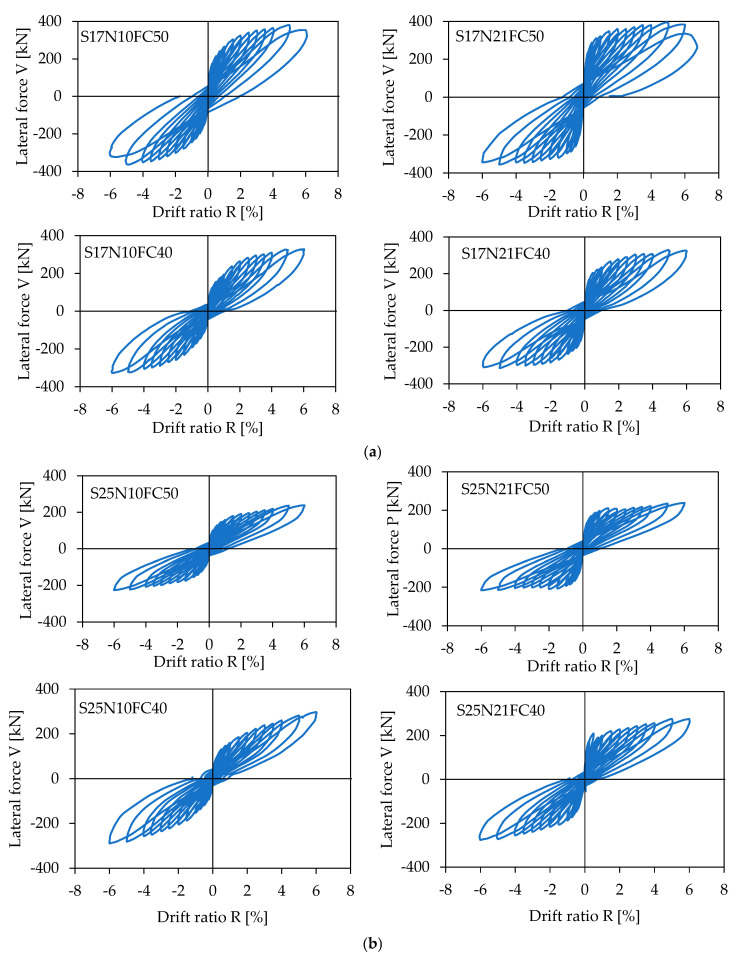
Measured V-R relationships: (**a**) specimens with a/D = 1.7; (**b**) specimens with a/D = 2.5.

**Figure 11:**
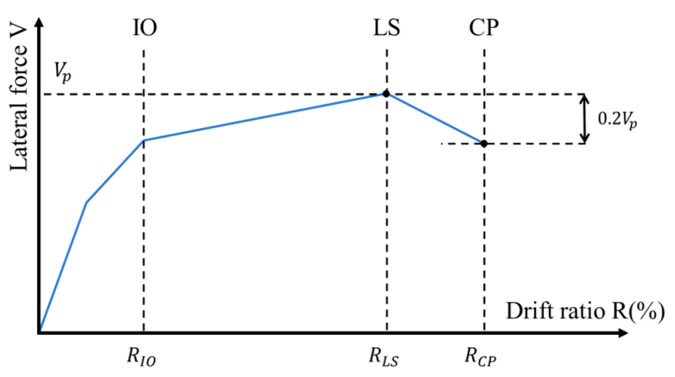
Idealization of damage states for ductile concrete structures and/or columns [16,17].

Figure 10 shows the measured lateral load (V) versus drift ratio (R) relationships for all specimens. As is apparent from Figure 9, all the specimens exhibited typical flexure-dominated behavior and their lateral resistances kept increasing up to the drift ratio of at least 4.0%. Given that the 4.0% drift ratio was twice as large as the threshold of the story drift ratio defining the so-called collapse prevention state for conventional ductile concrete structures (see Table 4 and Figure 11), the high resilience and robustness of the concrete columns reinforced by SBPDN rebars can be clearly and easily understood from Figure 10.

One can also see from Figure 10 that the higher the axial load level, the larger the lateral resistance capacity but the lower the tangential stiffness at large drift, which is an indicator measuring the restorability and resilience of concrete components inclusive of columns and walls. It is noteworthy that increasing the steel amount of SBPDN rebars could significantly enhance the tangential stiffness at large drift. On the other hand, the longer the shear span and the higher the concrete strength, the lower the tangential stiffness at large drift. The longer shear span increases the P-delta effect, and the higher concrete strength itself exhibits a steeper softening effect, both of which tend to reduce the lateral resistance of columns as the lateral deformation (drift) increases.

### 4.3. Residual Crack Widths and Residual Drift Ratios

**Figure 12:**
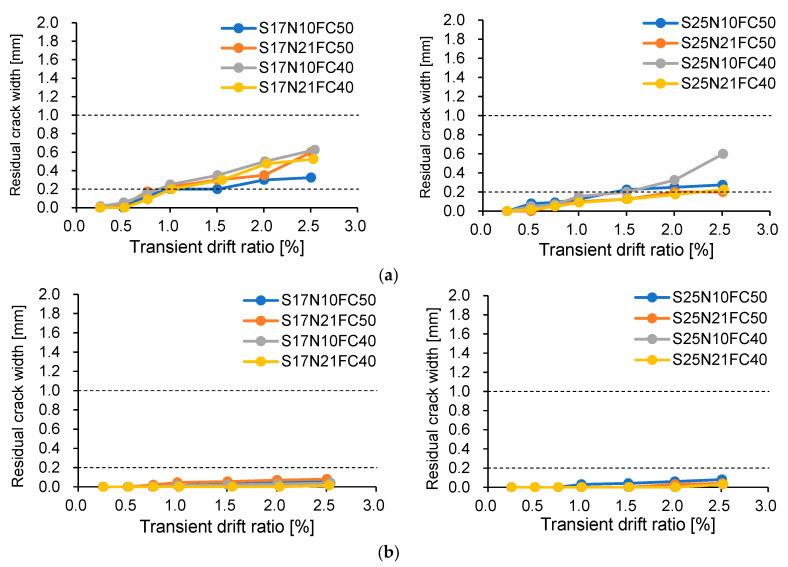
Measured residual crack widths: (**a**) flexural crack; (**b**) shear crack.

**Figure 13:**
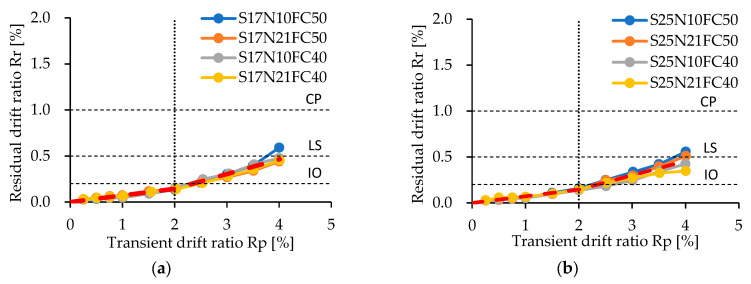
Measured residual drift ratios: (**a**) specimens with a/D = 1.7; (**b**) specimens with a/D = 2.5.

To further see the difference between ductile and resilient concrete columns, Figure 12 and Figure 13 compare the measured residual crack widths and residual drift ratios with the thresholds recommended in JBDPA guidelines [17] and FEMA guidelines [16], respectively, for ductile concrete structures. 

The measured residual crack widths shown in Figure 12 express the average of the crack widths measured in both push and pull directions. The crack widths were measured using a crack width ruler whose range varied from 0.03 mm to 2.5 mm. The two horizontal dotted lines superimposed in Figure 12 represent the crack widths defining the “operational” state and the lower “repairable” state prescribed in JBDPA guidelines, respectively. It is noted that the measured crack widths at the drift ratio of 2.5% are displayed only as reference because the crack widths measured at that drift ratio became unreliable due to the severe crushing of the cover concrete. 

As is apparent from Figure 12, the residual widths of flexural crack were mainly influenced by shear span ratio and much less than 1.0 mm after unloading from 2.0% drift ratio. One can also see that the residual widths of shear cracks were less than 0.1 mm and negligible even after unloading from 2.5%. These observations imply that resilient concrete columns have much higher repairability than ductile concrete columns.

In addition, one can see from Figure 13 that the measured residual drift ratios are kept under 0.5%, the threshold defining the minor damage state [16] even after unloading from a 4.0% drift ratio. The residual drift ratios increase almost linearly with the transient drift ratio (R_p_), and the increasing slope becomes a little steeper after R_p_ exceeds 2.0%, where the crushing of cover concrete became significant. While there is some scattering, among four experimental variables, only the shear span ratio exhibits a little influence on the slope after the 2.0% drift ratio. By performing linear regression analysis on the test results shown in Figure 13 and by further rounding off their coefficients, Equation (2) can be derived to estimate the residual drift ratio for resilient concrete columns with different shear span ratios.

(2) $$R_{r}=\left\{\begin{array}{l} 0.077\;R_{p},\;\;\;\;\;\;\;\;\;\;\;\;\;\;R_{p}<2.0\\ \left(0.172-0.007a/D\right)R_{p}-\left(0.206-0.017a/D\right),\;R_{p}\geq 2.0 \end{array}\right.$$

It is noted that the *R_r_* and *R_p_* in Equation (2) are in percentages. The ratio of the measured *R_r_* to the calculated one by Equation (2), which is superimposed in Figure 13 in red dashed lines, has a mean value of 1.00 and a standard deviation of 0.21, implying the high reliability and accuracy of Equation (2). 

Furthermore, the story drift ratio (R_s_) is related to the column drift ratio (R) in the form of R_s_ = (h_o_/H) R, where H and h_o_ are the story height and the clear story height, respectively. Therefore, under the same story displacement, the drift ratio of a column is larger than the story drift ratio and represents a stricter drift demand than the latter.

### 4.4. Fractal Dimensions and Drift Ratio-Based Evaluation Model 

**Figure 14:**
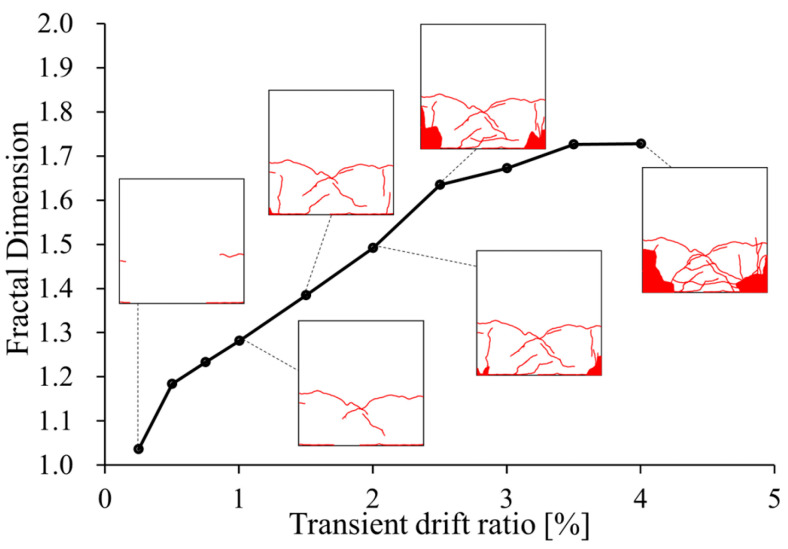
Example of propagation of crack pattern and corresponding FDs (S17N10FC50).

Figure 14 shows an example of the propagation of crack patterns and corresponding FDs calculated using the box-counting algorithm for specimen S17N10FC50. It is obvious from Figure 14 that the derived FD increases as the transient drift ratio increases and cracks propagate. The FD at the transient drift ratio of 0.25% is less than 1.1 and beyond 1.1 from a 0.5% drift ratio and upwards. Because the maximum residual crack width and residual drift ratio measured at the 0.5% drift ratio are less than 0.1mm and 0.06% (Figure 12 and Figure 13), respectively, implying that no repairing is needed [16,17], it can be recommended that only the FDs corresponding to transient drift ratios larger than 0.5% are meaningful from the perspective of post-earthquake damage assessment. 

**Figure 15:**
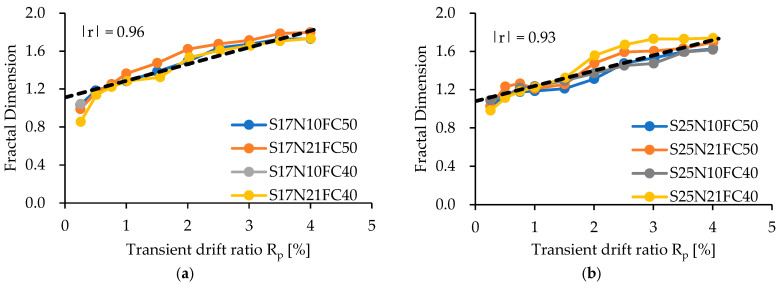
Fractal dimension (FD) versus transient drift ratio (R_p_) relationships: (**a**) specimens with a/D = 1.7; (**b**) specimens with a/D = 2.5.

To see the influence of experimental variables on FD, Figure 15 displays FD versus transient drift ratio relationships and Table 5 lists the FDs for all specimens. As is apparent from Figure 15, the FD increases as the transient drift ratio increases, and the larger the shea span ratio, the more gradual the increasing slope of the FD. The axial load level, concrete strength, and steel ratio of SBPDN rebars exhibit little influence on the varying trend of FDs. The FDs are not given in Figure 15 at larger drift ratios than 4.0%, because a 4.0% drift ratio is twice as large as the threshold defining the “collapse prevention” state for ductile concrete components and is large enough for resilient concrete columns from a practical viewpoint.

Variation in the derived FD is significantly observed at lower drift ratios than 0.5%, and the ascending slope of FDs along with drift ratios becomes gentle after the drift ratio of 0.5%. From the 0.5% drift ratio and upwards, the derived FDs exhibit very strong correlations with transient drift ratios. The black dashed straight lines superimposed in Figure 15 represent the best-fitting straight lines obtained by conducting linear regression analysis on the test results derived at 0.5% and larger drift ratios. The correlation coefficients of these two regression straight lines are as high as 0.96 and 0.93, indicating that the fractal dimension is a very useful and reliable indicator to quantify propagation and pattern changes in surface cracks for resilient concrete columns.

Considering that the maximum residual crack width and residual drift ratio measured at a 0.5% drift ratio are only 0.08 mm and 0.06%, respectively, and are much smaller than the thresholds defining the so-called minor damage state (see Table 4), from the viewpoint of practice and for simplicity, a single linear model can be developed to estimate FDs from transient drift ratios for resilient concrete columns in form of

(3) $$FD=\left(0.209-0.020\;a/D\;\right)R_{p}+\left(1.180-0.040\;a/D\right),\;\;R_{p}\geq 0.5\;(R_{p}\;\mathrm{in}\;\%)$$

Equation (3) is derived by combining the two expressions of the regression straight lines shown in Figure 15 into a unified expression so that it can take the influence of the shear span ratio into account. The ratio of experimentally derived FDs to the calculated ones by Equation (3) has a mean value of 1.00 and a standard deviation of 0.05, implying the high accuracy of the drift ratio-based FD model expressed by Equation (3). It is noteworthy that Equation (3) can also be used to extrapolate the FD at a smaller drift ratio than 0.5% and will give a conservative assessment of damage (see Figure 15).

### 4.5. Evaluation of Overall Seismic Damage by Fractal Dimension

As is obvious from Figure 15, the FD can efficiently quantify the crack patterns observed at any specific drift ratio for resilient concrete columns. However, the FD only estimates the degree of external damage appearing on the surface. It is not clear to what extent the FD can be used to quantify the degree of overall seismic damage, including the invisible damage caused by internal cracks and yielding of the steels placed in the columns. 

**Figure 16:**
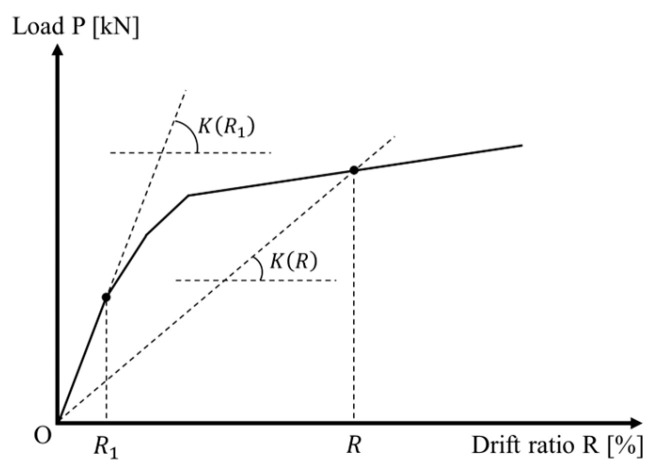
Definitions of initial stiffness and secant stiffness at different loads and drift ratios.

The relative stiffness loss (RSL) has been widely adopted in the field of earthquake engineering as an indicator measuring the global damage of concrete structures and/or components [10] and is expressed in form of

(4) $$RSL=1-\frac{K\left(R\right)}{K\left(R_{1}\right)}$$

where *K*(*R*) is the secant stiffness at any transient drift ratio *R*, and *K*(*R*_1_) is the initial stiffness, which can be approximated by the secant stiffness connecting the origin and the point of a 0.25% drift ratio at the backbone V-R curve of the test columns (see Figure 16). It is obvious that RSL = 0 and RSL = 1.0 represent the initial no-damage state and the complete collapse of a column, respectively.

The RSL was initially proposed by Cakmak et al. [49,50] and Miyamura et al. [51] to relate the global damage degree of a concrete structure to the change in its first natural period. Because the change in the first natural period of the structure is generally attributed to the accumulated damage, including undetectable internal micro-cracks of concrete and yielding of reinforcement, the RSL has also been applied to measure the overall damage for concrete components. However, the drawback of RSL is that it provides very little information on the crack distribution or pattern on a cracked surface, hence hindering the quantification of the repairability for damaged concrete components. An alternative index enabling structural engineers and/or decision-makers to quantify the overall damage degree is desirable.

**Figure 17:**
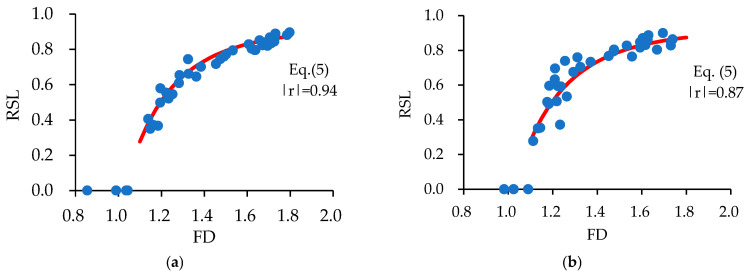
Relative stiffness loss (RSL) versus fractal dimension relationships: (**a**) specimens with a/D = 1.7; (**b**) specimens with a/D = 2.5.

Figure 17 shows the relationships between the derived FD and RSL. Large scatterings are only observed among the FDs derived at the initial transient drift ratio of 0.25%. As the FD is larger than 1.1, and the measured RSL exhibits a very strong correlation with the derived FD, Equation (5) is derived to correlate the RSL with the FD for resilient concrete columns.

(5) $$RSL=0.93-1.05{\left(FD\right)}^{-5},\;\;FD\geq 1.1$$

The red solid lines superimposed in Figure 17 express the best-fit line by Equation (5) along with respective correlation coefficients. The relatively high correlation coefficients indicate that Equation (5) can give a reliable and accurate prediction of the overall seismic damage degree for resilient concrete columns and that influences of experimental variables on the relationship between RSL and FD can be neglected. Furthermore, the ratio of the measured RSL to the calculated ones by Equation (5) has a mean value of 1.00 and a standard deviation of 0.10, confirming the high reliability and accuracy of Equation (5). 

It is noted that a lower limit is placed on the FD in applying Equation (5) because the FD less than 1.1 corresponds to the transient drift ratio less than 0.25 % (see Figure 15), and then, it is meaningless from the practical viewpoint of seismic damage assessment. 

## 5. Conclusions

Eight 300 mm-square concrete columns reinforced by SBPDN rebars were fabricated and tested under reversed cyclic lateral loading and constant axial compression to verify the usefulness of fractal analysis in quantifying the seismic damage degree for resilient concrete columns and simultaneously provide basic data on the conventional damage indicators such as residual crack width and drift ratio. The box-counting algorithm is implemented into Processing using Java script to perform fractal analysis of the crack patterns. Within the ranges of the tests, and from the experimental results and the fractal analysis described in this paper, the following conclusions can be drawn:

1. When reinforced by SBPDN rebars, concrete columns exhibit very high repairability and restorability up to a 4.0% drift ratio. The maximum residual crack width after unloading from a 2.0% drift ratio is kept less than 0.6 mm, while the maximum residual drift ratio after unloading from a 4.0% drift ratio is below 0.5%. Based on the test results, a bilinear model (Equation (2)) is derived to correlate the residual drift ratio with the transient drift ratio. The residual drift ratios calculated by Equation (2) show very good agreement with the measured ones.
2. The fractal dimension is a useful and efficient image-based indicator to quantify the propagation and pattern of cracks on the surface of resilient concrete walls. The derived FD increases as the transient drift ratio and damage degree increase. The larger the shear span ratio, the smaller the FD because the region of cracks did not expand with the shear span ratio, while the influence of the axial load level and steel ratio of SBPDN rebars on the FD is negligible. It is noteworthy that an FD less than 1.1 can be ignored because this FD represents the surface damage at the transient drift ratio of 0.125% and corresponds to the no-damage state.
3. Based on the test results, a transient drift ratio-based linear model (Equation (3)) is developed to calculate the fractal dimension. The correlation coefficients between the experimentally derived FDs and the calculated ones are as high as 0.961 and 0.93 for the columns with shear span ratios of 1.7 and 2.5, respectively. The ratio of experimentally derived FDs to the calculated ones by Equation (3) has a mean value of 1.00 and a standard deviation of 0.05, implying the high accuracy of the drift ratio-based FD model.
4. The measured FDs exhibit a very strong correlation with relative stiffness loss (RSL), a widely used indicator measuring the overall seismic damage of concrete components. Ignoring the FD less than 1.1, an empirical model (Equation (5)) is derived to evaluate the RSL from the FD. Very good agreement between the measured RSL and the calculated ones by Equation (5) indicates that the FD can also be utilized to assess the overall damage degree for resilient concrete columns.
5. The proposed models are concise and can be utilized to quantify the post-earthquake damage degree of damaged resilient concrete columns and pre-earthquake damage scenario prediction for those adopted in existing and/or new buildings. From the digital images of surface crack patterns taken on-site, after deriving the FD by the box-counting algorithm, the experienced transient drift ratio, the residual drift ratio, and the RSL of the damaged columns could be simply obtained using the proposed models in forms of Equations (2), (3) and (5). Meanwhile, from the transient drift ratios induced by an expected earthquake with a specific level of intensity, the proposed models can help decision-makers to perform reliable and quantitative judgment about the potential overall and local damage degree for new and/or existing concrete structures utilizing resilient concrete columns.

The above are the main findings obtained from this study. However, to make the proposed drift ratio-based damage assessment models more comprehensive and to widen their applicability to ductile concrete columns, follow-up tests and more experimental data are needed. The follow-up experiments are in progress, and the results will be reported in the near future. In addition, the FD derived from the crack patterns obtained by the finite element method should also be explored and compared with the experimentally measured ones, which can make it easy to obtain more and more basic data concerning crack distribution and promote the establishment of comprehensive damage assessment models for resilient and ductile concrete components.

## Figures and Tables

**Table 1:** Examples of the calculated *N*(*r*) by the box-counting algorithm.

r	$N\left(r\right)$
*H*	1
*H/2*	2
*H/4*	8
*H/8*	25
*H/16*	49
*H/32*	100
*H/64*	205
*H/128*	458
*H/256*	1170
*H/512*	4030

**Table 2:** Experimental parameters of the specimens.

Specimens	a/D	fc′ [MPa]	n	Longitudinal Reinforcement		Transverse Reinforcement	
				SBPDN Bar	p_g_ [%]	Configuration	p_w_ [%]
S17N10FC50	1.7	55.8	0.10	8-U15	1.51	Spiral D6@55 + Square hoop D6@100	0.60
S17N21FC50		52.0	0.21				
S17N10FC40		39.1	0.10				
S17N21FC40		40.2	0.21				
S25N10FC50	2.5	47.9	0.10				
S25N21FC50		49.1	0.21				
S25N10FC40		41.0	0.10	12-U15	2.30		
S25N21FC40		40.9	0.21				

a: shear span; D: depth of column section; f_c_′: concrete strength; n: axial load ratio (=N/bDf_c_′); N: axial load; b: width of column section; p_w_: steel ratio of transverse reinforcements.

**Table 3:** Mechanical properties of the steels used.

Notation	Grade	$E_{s}$ [kN/mm^2^]	$f_{sy}$ [N/mm^2^]	$\varepsilon_{y}$ [%]	$f_{su}$ [N/mm^2^]
D6	SD295A	197	420	0.24	503
U15	SBPDN1275	203	1410 *	0.89 *	1499

*E_s_*: Young’s modulus; *f_sy_* and *e_y_*: yield strength and strain; *f_su_*: tensile strength; * 0.2% offset yield strength and strain.

**Table 4:** Damage states of ductile concrete frames prescribed in current PBSD guidelines.

Guideline	Damage State	Damage Description	Transient Story Drift Ratio R_s_ [%]	Residual Story Drift Ratio R_r_ [%]	Residual Crack Width
FEMA [16]	IO ^(1)^	Minor	0.5	≤0.2	≤1.59 mm
	LS ^(2)^	Moderate	1.0	0.5	NA
	CP ^(3)^	Major	2.0	1.0	NA
JBDPA [17]	Operational	Minor	0.67	NA	~0.2 mm
	Repairable	Moderate	1.00	NA	1.0~2.0 mm
	Ultimate	Major	1.33	NA	>2.0 mm

Note: ^(1)^ Immediate occupancy; ^(2)^ life safety; ^(3)^ collapse prevention.

**Table 5:** The derived fractal dimensions.

Specimen	Transient Drift Ratio Rp [%]									
	0.25	0.50	0.75	1.00	1.50	2.00	2.50	3.00	3.50	4.00
S17N10FC50	1.04	1.18	1.23	1.28	1.38	1.49	1.63	1.67	1.73	1.73
S17N21FC50	0.99	1.16	1.25	1.36	1.47	1.62	1.68	1.71	1.79	1.80
S17N10FC40	1.04	1.15	1.19	1.20	1.33	1.45	1.50	1.64	1.69	1.71
S17N21FC40	0.85	1.14	1.22	1.28	1.32	1.53	1.61	1.66	1.71	1.73
S25N10FC50	1.02	1.14	1.18	1.19	1.21	1.31	1.48	1.53	1.60	1.63
S25N21FC50	1.03	1.23	1.26	1.21	1.26	1.48	1,59	1.60	1.63	1.70
S25N10FC40	1.09	1.13	1.22	1.24	1.29	1.37	1.45	1.47	1.59	1.62
S25N21FC40	0.98	1.11	1.18	1.22	1.32	1.56	1.67	1.73	1.73	1.74

## Data Availability

Data available on request due to restrictions.
